# CrAssphage as a Novel Tool to Detect Human Fecal Contamination on Environmental Surfaces and Hands

**DOI:** 10.3201/eid2608.200346

**Published:** 2020-08

**Authors:** Geun Woo Park, Terry Fei Fan Ng, Amy L. Freeland, Vincent C. Marconi, Julie A. Boom, Mary A. Staat, Anna Maria Montmayeur, Hannah Browne, Jothikumar Narayanan, Daniel C. Payne, Cristina V. Cardemil, Aimee Treffiletti, Jan Vinjé

**Affiliations:** Centers for Disease Control and Prevention, Atlanta, Georgia, USA (G.W. Park, T.F.F. Ng, A.L. Freeland, J. Narayanan, D.C. Payne, C.V. Cardemil, A. Treffiletti, J. Vinjé);; Atlanta Veteran Administration Medical Center, Atlanta (V.C. Marconi);; Emory University School of Medicine, Atlanta (V.C. Marconi); Texas Children’s Hospital, Houston, Texas, USA (J.A. Boom);; Cincinnati Children’s Hospital Medical Center, Cincinnati, Ohio, USA (M.A. Staat);; Cherokee Nation Assurance, Arlington, Virginia, USA (A.M. Montmayeur);; Oak Ridge Institute for Science and Education, Oak Ridge, Tennessee, USA (H. Browne)

**Keywords:** crAssphage, human fecal indicator, norovirus outbreak, environmental contamination, hand contamination, cruise ship, long-term care facility, viruses, enteric infections

## Abstract

CrAssphage is a recently discovered human gut–associated bacteriophage. To validate the potential use of crAssphage for detecting human fecal contamination on environmental surfaces and hands, we tested stool samples (n = 60), hand samples (n = 30), and environmental swab samples (n = 201) from 17 norovirus outbreaks for crAssphage by real-time PCR. In addition, we tested stool samples from healthy persons (n = 173), respiratory samples (n = 113), and animal fecal specimens (n = 68) and further sequenced positive samples. Overall, we detected crAssphage in 71.4% of outbreak stool samples, 48%–68.5% of stool samples from healthy persons, 56.2% of environmental swabs, and 60% of hand rinse samples, but not in human respiratory samples or animal fecal samples. CrAssphage sequences could be grouped into 2 major genetic clusters. Our data suggest that crAssphage could be used to detect human fecal contamination on environmental surfaces and hands.

Hygienic practices, including disinfection of environmental surfaces, are important to reduce exposure to pathogens that spread through fecal–oral transmission. Thus, monitoring of human fecal contamination and identifying the source of contamination is an important approach to prevent transmission of gastroenteritis viruses for which humans are the only natural host (e.g., human norovirus) (*1*). Culturable bacteria (e.g., *Escherichia coli*, *Enterococcus* spp., and *Bacteroides* spp.) are widely used as indicators to assess the presence of human fecal contamination of environmental waters (*2*–*5*). However, fecal indicator bacteria are not specific to human fecal contamination (*6*) and have a poor correlation with exposure risk to enteric viruses (*4*,*7*–*9*).

Over the past few decades, several viruses (e.g., human polyomavirus, Aichi virus, norovirus, and human adenovirus) have been studied as human fecal indicators for the detection of sewage-contaminated source and drinking water (*10*–*13*). Recently, both norovirus and adenovirus have been suggested as potential biomarkers of viral contamination to assess hygiene status and potential human health risk of contaminated surfaces and hands of affected persons (*4*,*12*,*14*–*17*). However, the detection of those viruses in indoor environments was relatively rare and inconsistent, making it difficult to estimate indoor hygiene and limiting their applicability for use in both industrial and regulatory settings (*12*,*14*–*17*).

Recently, a new DNA bacteriophage was discovered by computational analysis of publicly accessible human fecal metagenomics data and was named crAssphage, referring to the Cross-Assembly software that was used for its discovery (*18*). The single-stranded circular DNA genome is 97 kbp in size with 80 predicted open reading frames (ORFs) (*18*). Genetically, crAssphage are extremely heterogenous and can be grouped into at least 10 different genera (*18*,*19*). Various bacteria of the phylum *Bacteroidetes* have been proposed as the primary hosts of crAssphage, which was supported by recent findings that phage ΦCrAss001 from human feces could be isolated in *Bacteroides* (*20*). To date, crAssphage has primarily been detected in human stools and rarely in animals (*18*,*21*). In addition, crAssphage can be found at high levels in sewage throughout the year and correlate with the detection of fecal indicators (*E. coli*, enterococcus, human polyomavirus, and somatic coliphage), suggesting they could be used for monitoring human fecal pollution of water (*21*–*25*).

In this study, we aimed to validate the potential use of crAssphage to detect human fecal contamination on environmental surfaces and hands. We tested human stool samples, environmental swab samples, and hand rinse samples collected during norovirus outbreaks, as well as stool samples from persons without acute gastroenteritis (AGE) and saliva and nasal samples from humans with respiratory symptoms. To confirm the specificity of crAssphage for the human gut, we also tested fecal specimens from cats, rats, rhesus monkeys, and husbandry animals (cows, pigs, sheep, and horses).

## Materials and Methods

### Clinical Samples from Humans

In this study, we used archived fecal specimens that had been collected from patients with AGE from 5 norovirus outbreaks on cruise ships during 2015–2016 (n = 30) and from 12 norovirus outbreaks in long-term care facilities (LTCFs) in Oregon during 2013–2016 (n = 30) (*26*). In addition, we tested 22 vomitus samples from norovirus-positive patients as well as 43 saliva and 48 nasal swab samples from norovirus-negative patients. In addition, stool specimens from 2 cohorts of persons without AGE symptoms were included: adults >25 years of age who participated as non-AGE controls in a study to determine the incidence of norovirus in Veteran Affairs Medical Centers (VAMCs) (IRB approval no. 00091065) (*27*) and children <5 years of age who participated in the New Vaccine and Surveillance Network (IRB approval no. 6164) (*28*). Because stool samples from the norovirus outbreaks were categorized as public health nonresearch, human subject regulations did not apply.

### Fecal Samples from Animals

We obtained fecal samples from 3 laboratory animals (rhesus monkeys [n = 12], rats [n = 4], and cats [n = 2]) without diarrhea from archived collections at the Centers for Disease Control and Prevention (CDC) in Atlanta, Georgia. In addition, fecal DNA extracts from cows (n = 10), sheep (n = 10), horses (n = 10), and pigs (n = 30) were provided by Jeong Kwang Cheol at the University of Florida and Qiuhong Wang at Ohio State University.

### Environmental Swab and Hand Rinse Samples

We collected environmental swab samples at the time of disembarkation of 5 cruise ships (cruise ship A–E) that experienced norovirus outbreaks. We sampled hard surfaces, including toilet seats, toilet door handles, telephone handles, television remote controls, and door handles in cabins that had been occupied by passengers who had reported AGE symptoms by using macrofoam swabs (Puritan, https://www.puritanmedproducts.com) as described previously (*29*). On cruise ship B, we sampled the same environmental surfaces again 3 weeks later when no elevation of the number of AGE cases was reported; these samples are expressed as “B, follow up.” We collected all swab samples before standard surface cleaning procedures for each ship. We shipped swab samples on dry ice to CDC and stored them at −80°C until testing. We also included in this study archived hand rinse samples collected from 30 norovirus patients in LTCFs (*26*).

### Nucleic Acid Extraction and Real-Time PCR Detection of CrAssphage

**Figure 1:**
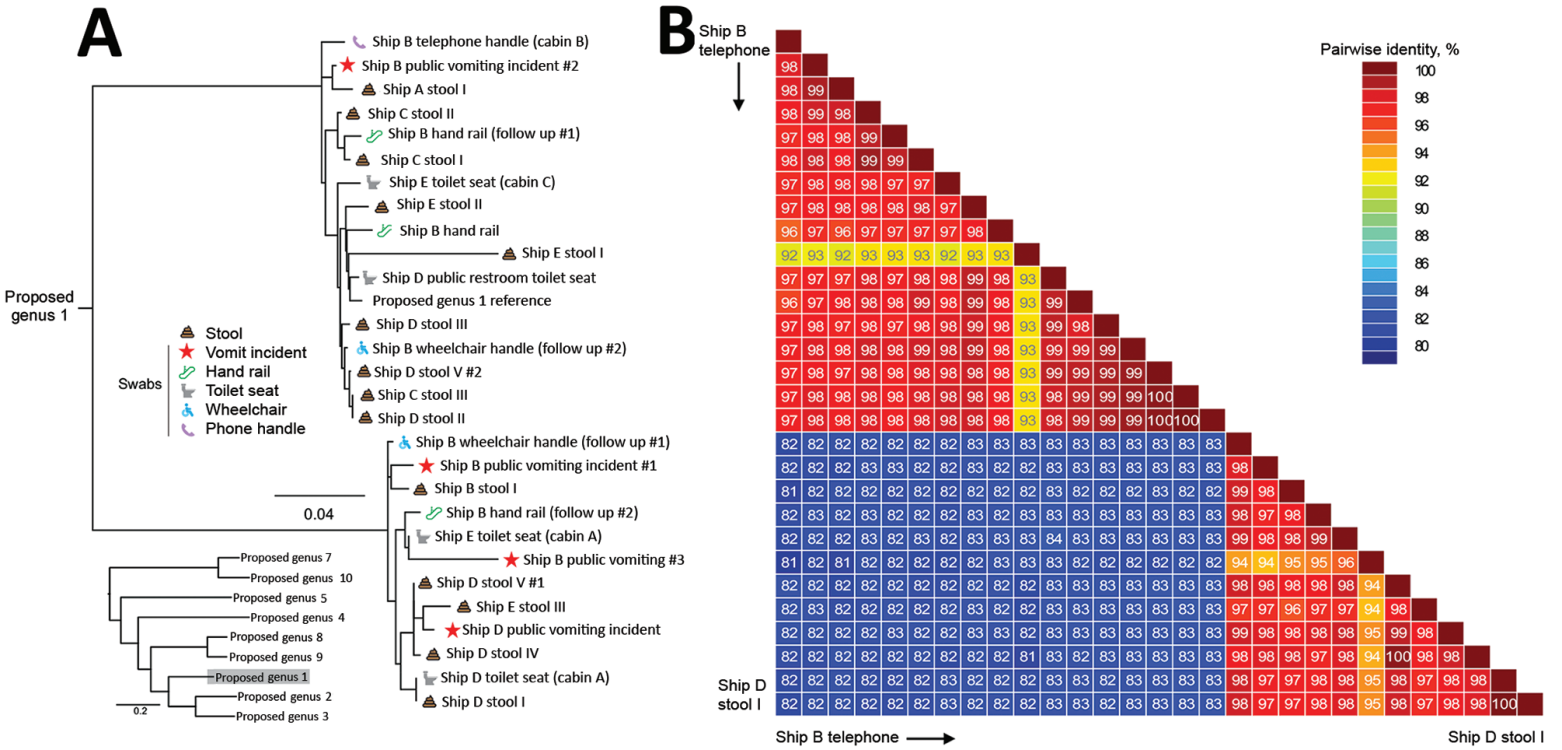
Phylogenetic relationships and pairwise sequence comparison of crAssphage strains from swab samples collected during norovirus outbreaks on cruise ships. A) Phylogeny of crAssphage on cruise ships, showing ship and source for each strain. Inset shows position of cruise ship strains among reference strains; scale bar indicates number of nucleotide changes between sequences. B) Color-coded pairwise identity matrix for crAssphage strains. Each cell includes the percentage identity among 2 sequences (horizontally to the left and vertically at the bottom). Key indicates pairwise identity percentages.

We extracted total nucleic acid from clinical samples, hand rinse samples, and swabs, as described previously (*26**,**29*; Appendix). We designed oligonucleotide primers and probes on the basis of conserved regions of the DNA polymerase gene (ORF00018) from 43 publicly available crAssphage strains by using the real-time quantitative PCR assay tool from Integrated DNA Technologies (https://www.idtdna.com) (*18*). Primer sequences had no more than 1 mismatch with the prototype CrAssphage genome (GenBank accession no. NC_024711). We tested extracted DNA from clinical and environmental samples by using TaqMan real-time PCR and the AgPath-ID One Step RT-PCR Kit (ThermoFisher Scientific, https://www.thermofisher.com ) on an Applied Biosystems 7500 platform (also ThermoFisher Scientific); the oligonucleotide primers (TN201/TN203) and probe (TN202) generated a 146-bp product (Table 1) (Appendix). We amplified the full-length ORF00018 (DNA polymerase) gene of a sample (ship E [stool II] in Figure 1) by using primers CrAssPol-F and CrAsspol-R (Table 1) to generate a 2,428-bp amplicon, as described previously (*30*). In each experiment, we included a 10-fold serial dilution (10^5.7^–10^0.7^ copies/3 μL) of this purified and quantified amplicon to generate a standard curve. The detection limit of the crAssphage real-time PCR was 0.7 DNA copies per PCR reaction. The average slope and deviation of the standard curves was 3.3626 + 0.0377 (r^2^ >0.9926). We calculated the concentration of crAssphage by converting cycle threshold values to DNA copies.

**Table 1:** Oligonucleotide primers and probe used for detection and typing of crAssphage

Primer or probe	DNA sequence	Position*	Description
CrAssPol_F†	5′-CGG CGG GTT AAT CAA AAT AGA A-3′	8907–8928 (flanking pol)	Forward primer conventional PCR
CrAssPol_R†	5′-GCG GAG AAC CCC ATT TAT TAA TAA G-3′	11334–11310 (flanking pol)	Reverse primer conventional PCR
TN201	5′-ATG TWG GTA RAC AAT TTC ATG TAG AAG-3′	10919–10945 (within pol)	Forward primer real-time PCR
TN203	5′-TCA TCA AGA CTA TTA ATA ACD GTN ACA ACA-3′	11111–11082 (within pol)	Reverse primer real-time PCR and typing PCR
TN202	FAM-5-ACC AGC MGC CAT TCT ACT ACG AGH AC-3-BHQ1	11079–11054 (within pol)	Probe real-time PCR
JP1crasF	5′-TAA AAC TAC WAT TTA TAG AGT TAA TAA AGA TGC STT TAG T-3′	10023–10062 (within pol)	Forward primer typing

*The sequence position was determined against a crAssphage sequence from GenBank (accession no. NC_024711). †Liang et al. (*30*).

### DNA Sequencing of crAssphage

**Figure 2:**
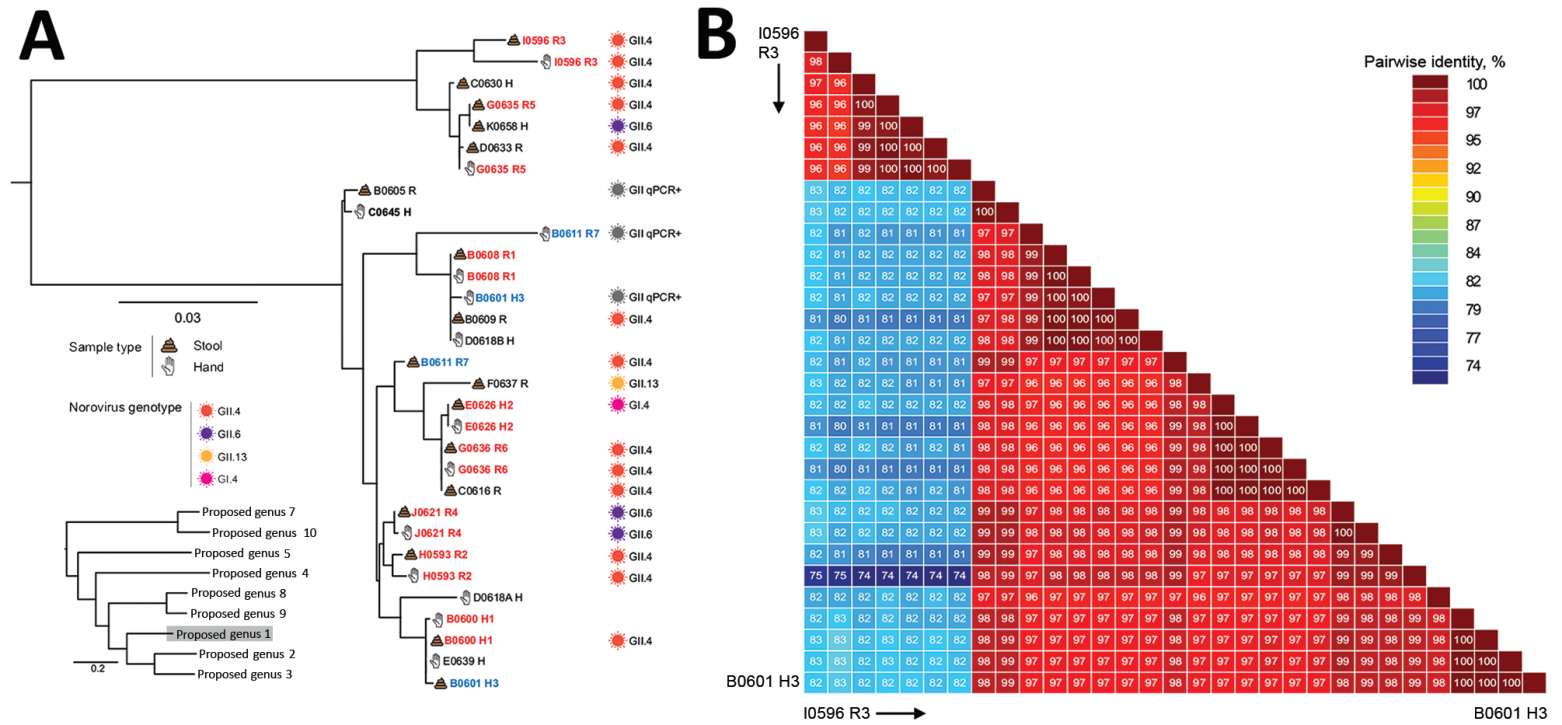
Phylogenetic relationships and pairwise sequence comparison of crAssphage strains from hand rinse samples collected during norovirus outbreaks at long-term care facilities. A) Phylogeny of crAssphage strains. Strain identification includes facility (A–J), strain number, and human source (H, healthcare worker; R, resident) for each isolate. Sample source and genotypes are indicated. Red strain names indicate that both hand and stool sample are genetically related, blue strain names that paired hand and stool samples are genetically distinct. Black strain names indicate hand or stool sample pairs that tested negative for crAssphage. Inset shows position of long-term care strains among reference strains; scale bar indicates number of nucleotide changes between sequences. (B) Color-coded pairwise identity matrix for crAssphage strains. Each cell includes the percentage identity among 2 sequences (horizontally to the left and vertically at the bottom). Key indicates pairwise identity percentages

To enable sequencing, we amplified DNA from real-time PCR–positive samples by using oligonucleotide primers JP1crasF/TN203 (Table 1) to generate a 1,089-bp PCR amplicon (Appendix). We sequenced the purified PCR products by using 500-cycle (2 × 250-bp paired-end) MiSeq Reagent Kit (Illumina, https://www.illumina.com). After filtering and trimming raw sequence reads, we assembled contigs by using the de novo assembler SPAdes 3.7.0 (http://cab.spbu.ru/software/spades) (Appendix). We analyzed assembled crAssphage amplicon sequences by read mapping and gene annotation using Geneious 11.1.2 (Biomatters, https://www.geneious.com), as described previously (*31*). The GenBank accession numbers for the strains sequenced in this study are MT475797–824 (Figure 1) and MT475766–96 (Figure 2). 

### Phylogenetic and Sequence Analyses

We generated multiple sequence alignment of crAssphage sequences by using MUSCLE (*32*) and constructed maximum-likelihood phylogenetic trees by using PhyML (*33*). We used the best nucleotide substitution model analyzed by Smart Model Selection based on the Bayesian information criterion (*34*) and calculated pairwise nucleotide identity (NI) between sequences by using the Sequence Demarcation Tool (*35*).

### Data Analysis

We determined sensitivity, specificity, and predictive values of crAssphage on norovirus co-infection (or co-contamination) as described previously (*36*). We performed log transformation, followed by the Wilcoxon rank-sum test, to compare the median crAssphage concentration (log_10_ genomic copies per sampled object for surface and hand rinse sample or per gram for stool) between different comparison groups. We used SPSS Statistics 21 (IBM, https://www.ibm.com) for statistical calculations and considered differences with p values <0.05 to be statistically significant (*37*).

## Results

### Detection of crAssphage in Human and Animal Samples

Overall, we detected crAssphage in 71.4% of human stool samples, including 42 (70.0%) of the 60 stool samples collected from 17 norovirus outbreaks, 46 (48%) of the 96 stool samples from adults >25 years of age without AGE symptoms and 53 (68.8%) of the 77 stool samples from children <5 years of age with AGE symptoms (Table 2). Specifically, 23 (76.7%) of the 30 stool samples from the 5 norovirus outbreaks on cruise ships tested positive, and 19 (63.3%) of 30 stool samples collected from the 12 norovirus outbreaks in LTCFs tested positive (Table 2). Thirty-nine (65.0%) of the 60 stool samples tested positive for both crAssphage and norovirus. The median concentration of crAssphage per gram of stool ranged from 5.9 (range 2.8–8.9) log_10_ genome copies in samples from norovirus outbreaks to 8.1 (range 3.1–10.3) log_10_ genomic copies in samples from adults without AGE symptoms and 8.4 (range 4.1–10.1) log_10_ genomic copies in samples from children <5 years of age without AGE symptoms. All vomitus samples from patients in norovirus outbreaks as well as saliva and nasal swab samples from children with respiratory symptoms tested negative for crAssphage. We did not detect crAssphage in any of the 78 fecal samples from animals.

**Table 2:** Prevalence of crAssphage in stool samples from norovirus outbreaks on cruise ships and in long-term care facilities and healthy controls without acute gastroenteritis

Setting (no. samples)	Age, y, (range)	% CrAssphage (no. positive/no. tested)	crAssphage titer (range)*
Cruise ship voyages† (5)	65.5 (29–88)	76.7% (23/30)	4.5 (3.2–8.9)
Long-term care facilities‡ (12)	63.5 (18–87)	63.3% (19/30)	5.4 (2.8–8.9)
Adults without acute gastroenteritis (96)	59.0 (28–83)	48% (46/96)	8.1 (3.1–10.3)
Children without acute gastroenteritis (77)	1.1 (0.2–5.0)	68.8% (53/77)	8.4 (4.1–10.1)

*log_10_ genomic copies per gram of stool sample. †This study. ‡Park et al. (*26*).

### Detection of crAssphage on Environmental Surfaces on Cruise Ships

We collected a total of 201 swab samples from frequently touched surfaces on 6 cruise ship voyages (5 cruise ships [A–E]) during norovirus outbreaks and 1 cruise ship (B, follow up) 3 weeks after a norovirus outbreak). We detected crAssphage DNA in 113 (56.2%) of the swab samples. The rates for each individual cruise ship were as follows: 15.2% (5/33) (cruise ship A); 84.8% (28/33) (cruise ship B); 62.3% (19/31) (cruise ship C); 44.7% (17/38) (cruise ship D); 72.7% (24/33) (cruise ship E); and 60.6% (20/33) (cruise ship (B, follow up).

#### On Surfaces in Cabins of Norovirus-Positive Patients

A total of 47 (58.8%) of 80 swab samples tested positive for crAssphage; median concentration was 2.5 (0.8–5.6) log_10_ genomic copies per surface (Table 3). Remote controls had the highest crAssphage contamination (87.5%), followed by toilet seats (68.5%), which had the highest crAssphage titer, 3.3 log_10_ (1.2–5.6) log_10_ genomic copies per seat. Of the 80 swab samples, 29 (36.3%) tested positive for norovirus; 11 of those samples also tested positive for crAssphage. The positive predictive value (PPV) of norovirus co-infection of crAssphage-positive samples was 38.0%; the negative predictive value (NPV) was 29.4% (Table 4). Compared with all surfaces sampled, toilet seats (56.3%) in the cabins from AGE-positive passengers had the highest norovirus contamination, with an average concentration of 5.5 (3.1–7.4) log_10_ genomic copies per surface.

**Table 3:** Prevalence of crAssphage and human norovirus on environmental surfaces on 5 cruise ships with norovirus outbreaks

Sampled objects†	CrAssphage			Norovirus	
	No. positive/total no. (%)	Concentration* (range)		No. positive/total no. (%)	Concentration* (range)
Cabins of norovirus-positive patients					
Toilet seats^P^	11/16 (68.5)	3.3 (1.2–5.6)		9/16 (56.3)	5.5 (3.1–7.4)
Toilet door handles^M^	7/16 (43.75)	2.3 (1.0–3.1)		5/16 (31.3)	5.1 (3.7–5.7)
Telephone handles^P^	9/16 (56.4)	2.4 (1.7–3.5)		3/16 (18.8)	4.9 (4.9–5.5)
Remote control surfaces^P^	14/16 (87.5)	2.6 (1.4–4.1)		5/16 (31.3)	3.6 (2.9–5.1)
Cabin door handles^M^	6/16 (37.5)	2.0 (0.8–3.4)		6/16 (37.5)	4.4 (3.1–6.0)
Overall	47/80 (58.8)	2.5 (0.8–5.6)		29/80 (36.3)	4.8 (3.1–7.4)
Public area (passenger area)					
ATM buttons^P^	2/3 (66.6)	2.8 (2.4–3.4)		1/3 (33.3)	1.8
Menu folder^L^	2/5 (40)	1.9(1.7–2.1)		1/5 (20.0)	4.9
Condiment containers^M^	2/5 (40)	2.2(2.1–2.3)		0/5 (0)	
Buffet utensils^M^	1/2 (50)	1.6		0/2 (0)	
Ice cream handle^P^	1/4 (25.0)	2.2		0/3 (0)	
Casino chips^P^	3/5 (60)	2.7 (2.1–2.8)		0/5 (0)	
Medical center clipboards^P^	3/5 (60)	2.3 (2.1–2.5)		1/5 (20)	5.1
Gift shop register touch screens^P^	3/5 (60)	3.0 (2.7–3.0)		0/5 (0)	
Youth center toys^P^	2/4 (50.0)	2.1 (2.1–2.2)		0/4 (0)	
Atrium hand rails^W^	2/5 (40.0)	3.0 (2.1–3.9)		1/5 (20)	4.1
Internet café keyboards^P^	3/5 (60)	1.8(1.8–2.5)		0/5 (0)	
Wheelchair handle rests^P^	2/5 (25)	2.9 (2.0–3.8)		0/5 (0)	
Hand contact surfaces exposed to public vomiting incident^M^	2/4 (50)	3.2 (2.8–3.5)		1/4 (25)	4.3
Toilet seat surfaces in public rest room^P^	3/3 (100)	4.7 (2.4–4.7)		0/3 (0)	0
Overall	21/64 (51.5)	2.4 (1.6–3.2)		5/64 (7.8)	3.3 (1.8–5.1)
Public area (crew area)					
Time clock machines^P^	3/5 (60)	2.2 (2.1–2.5)		0/5 (0)	
Edges of trolley for dirty linens^P^	5/5 (100)	2.3 (1.8–3.2)		0/5 (0)	
Elevator buttons in food service areas^P^	3/5 (60)	2.3 (2.1–3.1)		0/5 (0)	
Computer keyboard^P^	2/5 (40)	2.3 (2.0–2.5)		0/5 (0)	
Countertop surfaces in crew smoking room^W^	2/5 (40)	2.3(2.1–2.5)		0/5 (0)	
Overall	15/25 (60)	2.3 (1.8–3.2)		0/25 (0)	

^1^log_10_ genomic copies per sampled object. ^2^Each superscripted character indicates surface material of sampled object as follows: P, plastic; M, metal; W, wood; and L, leather.

**Table 4:** Sensitivity, specificity, and predictive values of norovirus co-contamination on crAssphage positive environmental surfaces and hand rinse samples*

Setting	Sample type	Source (no. samples)	Sensitivity, %	Specificity, %	PPV, %	NPV, %
Cruise ship†	Swab sample	Case cabin (80)	23.4	45.5	38.0	29.4
		Public area (64)	9.5	93.0	40.0	67.8
		Overall (144)	19.1	72.4	38.2	50.0
LTCFs‡	Hand rinse sample	Resident (15)	72.7	25.0	72.7	25.0
		HCW (15)	28.6	87.5	66.7	58.3
		Overall (30)	55.6	66.7	71.4	50.0

*HCW, healthcare worker; LTCF, long-term care facility; NPV, negative predictive value; PPV, positive predictive value. †This study ‡Park et al. (*26*).

#### On Surfaces in Public Areas

Of all surfaces in public areas on cruise ships that were sampled, 51.5% (21/64) tested positive for crAssphage; median concentration was 2.4 (1.6–3.2) log_10_ genomic copies per surface. Five surfaces in public areas (casino chips, a medical center clipboard, gift shop register touch screens, surfaces that were exposed to a public vomiting incident, and edges of a trolley for dirty linen) had contamination of >50%. Only 5 surfaces, including a menu folder (cruise ship A), a medical center clipboard (cruise ship A), hand contact surfaces in the public vomiting incident location (cruise ship A), hand rails in the atrium (cruise ship B), and ATM buttons (cruise ship C), tested positive for human norovirus; titers were 1.8–5.1 log_10_ genomic copies per surface. Two of the 5 norovirus-positive surfaces (a medical center clipboard and a handrail in the atrium) also tested positive for crAssphage. PPV for norovirus co-contamination of crAssphage-positive surfaces was 40.0%; NPV was 67.8.

#### Three Weeks after a Norovirus Outbreak

Seven (46.7%) of the 15 swab samples collected from cabins on cruise ship B that had been occupied by patients with AGE 3 weeks earlier tested positive for crAssphage, whereas 7 (38.9%) of the 18 surfaces in public areas tested positive. Compared with results from the same cruise ship immediately after the outbreak 3 weeks earlier, the number of crAssphage-positive surfaces decreased from 12 to 7 in public areas of cruise ship B. However, swabs from 6 surfaces (ATM buttons, a buffet utensil, a medical center clipboard, atrium handrails, a wheelchair handle rest in passenger areas, and smoking bar countertop surfaces in the crew smoking room) tested positive for crAssphage again. Two crAssphage-positive swab samples collected from a handrail (ship B handrail, follow-ups 1 and 2) and a wheelchair (ship B wheelchair, follow-ups 1 and 2) on cruise ship B contained multiple crAssphage sequences (Figure 1). These sequences were genetically distinct from those detected during the norovirus outbreak on the same cruise ship 3 weeks earlier (voyage B), suggesting not persistence of previous fecal material but more recent contamination with human fecal matter. In contrast, norovirus contamination on surfaces decreased from 24.2% (8/33) during the outbreak to 3.0% (1/33) 3 weeks later.

### Contamination of Hands with crAssphage during Norovirus Outbreaks in LTCFs

In total, 18 (60.0%) of 30 hand rinse samples tested positive for crAssphage, including samples from 7 healthcare workers (HCWs) and 11 residents. Both hand rinse and stool samples from 15 norovirus patients (4 HCWs and 11 residents) tested positive for crAssphage (Appendix Table). In a previous study, we reported that 14 (46.7%) of 30 hand rinse samples tested positive for norovirus, including 3 HCWs and 11 residents (*26*). Overall, 2 (28.6%) of 7 crAssphage-positive hand rinse samples from HCWs and 8 (72.7%) of 11 from residents tested positive for human norovirus. The PPV was 72.7% and the NPV 25.0% for co-contamination of crAssphage-positive hands of residents with norovirus, whereas for HCWs the PPV was 66.7% and NPV 58.3%.

### Sequence and Phylogenetic Analysis of crAssphage

Of the 42 PCR-positive stool samples, 30 (71.4%) were successfully sequenced and the titer of the remaining 12 samples was too low. Sequences from several crAssphage were identical (e.g., ship Dstool V# 1 and ship D stool IV, and ship C stool III and ship D stool II) (Figure 1). Overall, crAssphage sequences detected in samples from cruise ships rarely clustered closely together (Figure 1), whereas crAssphage from LTCFs were more closely related, with near-identical sequences (99%–100% NI) (Figure 2).

From the cruise ship outbreaks, 13 (56.5%) of the 23 crAssphage-positive stool samples and 10 (8.8%) of 113 crAssphage-positive swab samples were sequenced successfully. Phylogenetic analysis showed that crAssphage sequences could be grouped in 2 genetic clusters within proposed genus 1 (*19*) (Figure 1). Several samples contained multiple crAssphage sequences, including multiple stool samples with >1 sequence (ship D), swab samples from a wheelchair and a handrail each containing 2 sequences (ship B, follow up), and a swab sample from a public vomiting event (ship B) containing 3 different crAssphage sequences.

From the crAssphage in LTCFs, 17 (89.5%) of 19 stool samples and 13 (72.2%) of 18 positive hand rinse samples were successfully sequenced. The sequences could be grouped in 2 clusters that had <83% NI (Figure 2, panel A). Within each cluster, crAssphage was genetically diverse, with pairwise NI ranging from 96% to 100% (Figure 2, panel B). Identical crAssphage sequences were detected in paired hand rinse and stool samples from 5 persons (3 residents [G0636, B0608, and J0621] and 2 HCWs [E0626 and B0600]) (Figure 2). Among those persons, 2 residents [G0636, and J0621] had identical norovirus sequences in paired hand and stool samples as well. In contrast, crAssphage sequences in stool samples from 2 patients (resident D0611 and HCW B0601) did not match their corresponding hand rinse sample. Also, norovirus was detected only in the hand sample, not a stool sample, from HCW B0601. A single crAssphage sequence was detected on hands from 2 HCWs (D0645 and C0639), and 2 genetically different crAssphages were detected on the hand from 1 HCW (D0618), whereas all stool samples from these 3 HCW tested crAssphage negative. 

The median concentration of crAssphage in the 30 stool samples that could be sequenced was 6.5 (range 2.8–8.9) log_10_ genomic copies per gram of stool, compared with 3.9 (range 3.2–6.6) copies for samples that could not be sequenced (p<0.001). Viral load of crAssphage detected in environmental samples that could be sequenced was 3.5 (2.0–5.4) log_10_ genomic copies (n = 10), compared with 2.3 (range 0.6–5.6) copies (n = 103) for samples that could not be sequenced.

## Discussion

We detected crAssphage in >60% of stool samples from patients with AGE during norovirus outbreaks as well in at least half of stool samples from healthy populations but not in other clinical materials (vomitus, saliva, or nasal rinse) or fecal specimens from animals. The high prevalence of crAssphage on surfaces and hands in norovirus outbreak settings suggests that these phages can be used as an indicator to monitor human fecal contamination of environmental sources other than sewage-contaminated water (*21*–*25*). CrAssphage contamination was also frequently found on environmental surfaces in public areas of cruise ships both during and after norovirus outbreaks, suggesting a potential role of crAssphage in monitoring fecal contamination on surfaces in common settings that could be targeted for enhanced cleaning and disinfection practices.

CrAssphage can be classified into 4 subfamilies, which can be further divided into 10 candidate genera (*18*,*19*). In agreement with data from previous studies (*19*,*38*), we found genetically different phages in stools and environmental surfaces from different norovirus outbreaks. The extreme genetic diversity of crAssphage could help to determine possible contamination sources. For example, crAssphage strains in stool samples collected from the same LTCFs during outbreaks displayed a strong degree of interpersonal variation. Thus, identical crAssphage sequences found in stool and hand rinse samples of the same person suggest self-contamination, whereas different sequences suggest possible contamination with fecal material from someone else. On the basis of these assumptions, we concluded that the hands of most LTCF residents were frequently self-contaminated, whereas the hands of HCWs were more likely cross-contaminated, either by contact with frequently touched environmental surfaces or by assisting norovirus patients, highlighting the need to strictly adhere to hand hygiene practices and to take additional contact precautionary measures during norovirus outbreaks.

This study has several limitations. First, although we designed our PCR assay to detect crAssphage based on a larger number of sequences than were used in previous studies (*21*,*23*,*30*,*39*), only viruses from 1 of the 10 recognized crAssphage genera were detected, suggesting that crAssphage from other genera would likely have been missed. Second, because gastroenteritis viruses such as norovirus are often transmitted through vomitus or aerosols, use of crAssphage during outbreaks might be limited (*40*–*42*). Finally, because crAssphage assay was not validated with other domestic animals that share human-occupied spaces (e.g., dogs), nonhuman fecal contamination could not completely be ruled out.

CrAssphage are strongly correlated with bacterial species related to *Bacteroidetes* but are not associated with diarrheal disease in adults (*38*,*43*). Thus, the presence of crAssphage does not correlate with norovirus contamination but rather with human fecal contamination. Detection of crAssphage on environmental surfaces might help to better assess exposure risk for human norovirus in public areas (e.g., on cruise ships) as well as help to identify frequently touched surfaces that are often fecally contaminated as key sites for enhanced cleaning practices. Sequence analysis of crAssphage in paired hand rinse and stool samples provided laboratory evidence that hands of several persons were likely cross-contaminated with fecal material from other patients, suggesting that crAssphage can be used as a tool to monitor fecal contamination patterns. Because we did not test crAssphage contamination before or after cleaning of environmental surfaces, or assess hand hygiene practices performed by the staff in the LTCFs during the norovirus outbreaks, we recommend additional studies to guide prevention measures, such as enhanced cleaning (e.g., use of the US Environmental Protection Agency’s registered products of list G).

Appendix Additional information about CrAssphage as a novel tool to detect human fecal contamination on environmental surfaces and hands.
