# Supplementary material for: CrAssphage as a Novel Tool to Detect Human Fecal Contamination on Environmental Surfaces and Hands
Source: Emerg Infect Dis. 2020 Aug;26(8):1731–9. doi: 10.3201/eid2608.200346 (PMC7392416; doi:10.3201/eid2608.200346)
Supplement: Appendix — Additional information about CrAssphage as a novel tool to detect human fecal contamination on environmental surfaces and hands. [file 20-0346-Techapp-s1.pdf]

Article DOI: https://doi.org/10.3201/eid2608.200346

CrAssphage as a Novel Tool to Detect Human Fecal Contamination on Environmental Surfaces and Hands

Appendix

Materials and Methods

Total Nucleic Acids Extraction, Purification, and Concentration

Stool Samples

200 mg of stool samples and 5 µL of coliphage MS2 (10^6 pfu/µL) were suspended in a 200 µL of PBS (Phosphate-buffered saline) pH 7.2 and centrifugated at 10,000 × g for 2 min at room temperature to remove solid particles. 100 µL of supernatant was collected, then mixed with an equal volume of a UNEX lysis buffer (Microbiologics, St Cloud, MN) to extract nucleic acid, then purified using mini column (Omega Biotech, Norcross, GA) as previously described (*1*), and nucleic acid was eluted into 50 µL elution buffer.

Swab Samples

Swab samples were placed in a 15-mL centrifuge tube and 5 mL UNEX lysis buffer (Microbiologics, St Cloud, MN, USA) and 5 µL of coliphage MS2 (10^6 pfu/µL) were added (*1*). Briefly, 5 mL of 100% ethanol was added and after mixing by vortexing, the UNEX/ethanol mixture was loaded on a midi column (Omega Bio-Tek, Norcross, GA) and after a wash with 500 ul of 70% ethanol, nucleic acid was eluted into 250 µL DNA suspension buffer (Teknova, Hollister, CA). The nucleic acid was further concentrated to 50 µL using an RNA spin column (Omega Bioteck, Norcross, GA).

Hand Rinse Samples

Archived hand-rinse samples (50 mL) were thawed and centrifuged at 10,000 × g for 30 min at 4°C to separate supernatant from solid particles. Polyethylene glycol (PEG; molecular weight of 7,000 to 9,000), NaCl, and bovine serum albumin (molecular grade) (ThermoFisher

Scientific, Waltham, MA) were added to the supernatant to a final concentration of 8%, 0.3 M and 1%, respectively (*2*,*3*). The mixture was stirred slowly for 4 h at room temperature and the virus-containing pellet was collected by centrifugation at 10,000 × g for 30 min at 4°C. The pellet was re-suspended in 1 ml of phosphate buffer solution (0.01M, pH 7.4) and transferred into a 1.5 mL Eppendorf tube and centrifuged at 17,949 × g for 1 min. The supernatant was collected was transferred to a new tube and mixed with an equal volume of UNEX lysis buffer (Microbiologics) and 5 µL of coliphage MS2 (10^6 pfu/µL). The extracted viral nucleic acid was then purified using midi-columns (Omega Biotech, Norcross, GA) and concentrated to 50 µL using RNA clean and concentrators (Zymo Research, Irvine, CA, USA).

Construction of a 2,428 bp Amplicon for quantifying crAssphage

The full-length ORF00018 (DNA polymerase) gene of a sample (ship E [stool II] in Figure 1) was amplified using primers CrAssPol-F and CrAsspol-R (Table) to generate a 2,428-bp amplicon using Phusion High Fidelity DNA polymerase (ThermoFisher Scientific) and 5 pmol of forward and reverse primers as previously described (*4*). (Table 1, https://wwwnc.cdc.gov/EID/article/26/8/20-0346-T1.htm) PCR reaction conditions included an initial denaturation step at 98°C for 30s, followed by 40 cycles of 98°C for 10s, 50°C for 30s, and 72°C for 2.5 min, and a final extension step at 72°C for 10 min. The PCR product was 2,428 bp in length and was gel-purified using a gel extraction kit (Qiagen, Germantown, MD) and quantified using a Nanodrop spectrophotometer. Purified PCR products were used to make a standard curve for quantifying crAssphage.

CrAssphage Real-Time PCR Conditions

Extracted nucleic acid from clinical and environmental samples was tested by TaqMan real-time PCR using the AgPath-ID One Step RT-PCR Kit (ThermoFisher Scientific) on an ABI 7500 platform with 0.6 pmol of each oligonucleotide primers (TN201/TN203) and 0.3 pmol of FAM-labeled probe (TN202) (Table 1, https://wwwnc.cdc.gov/EID/article/26/8/20-0346-T1.htm). PCR amplification was performed after an initial denaturation step at 95°C for 30s, for 40 cycles at 95°C for 10s and 57°C for 30s. To determine the limit of detection and limit of quantification of this assay, the 2,428 bp purified PCR product (see above) was serially diluted from $10^{5.7}$ to $10^{0.1}$ copies/3 µL and each dilution was tested as described above. The limit of detection of the assay was 0.7 DNA copies per reaction and the limit of quantification was 2.5–

2.6 DNA copies per gram of animal or human fecal sample and 1.9 DNA copies per reaction for the swab and hand rinse samples.

Conventional PCR for typing of crAssphage

A 1,089-bp region of the polymerase gene of CrAssphage was amplified using oligonucleotide primers JP1crasF/TN203 and Phusion High Fidelity DNA polymerase (ThermoFisher Scientific) according manufacturers' protocol (Table 1, https://wwwnc.cdc.gov/EID/article/26/8/20-0346-T1.htm)PCR conditions included an initial denaturation step at 98°C for 30s followed by 40 cycles of 98°C for 10s, 48°C for 30s, and 72°C for 1.5 min, followed by a final extension at 72°C for 10 min.

Norovirus realtime RT-PCR for detection and hemi-nested PCR for genotyping

GI and GII norovirus were detected by reverse transcription–TaqMan real-time polymerase chain reaction (RT-qPCR) assays (*1*). To quantify the results into viral RNA copies, standard curves of GI.7 and GII.12 RNA transcripts were included in each run. To genotype norovirus positive samples, nucleic acid was amplified by hemi-nested PCR (*1*). PCR products were visualized on a 2% Seakem-ME agarose gel (Lonza, Allendale, NJ) containing Gel Red (Biotium, Fremont, CA) and purified using the QIAquick PCR purification kit (Qiagen, Germantown, MD) and Sanger sequenced using the PCR primers (Eurofins MWG Operon, Louisville, KY). Sequences were typed by comparing to norovirus reference sequences (*5*).

Next Generation Read Processing

Purified DNA amplicons of a 1,089-bp region of the polymerase gene of CrAssphage were sequenced using MiSeq Reagent Kit (Illumina, https://www.illumina.com), and sequence reads were filtered, trimmed and assembled as follows. Specifically, an in-house bioinformatics pipeline (*6*) was used to process raw FASTQ data as follows: host removal using Bowtie 2 v2.3.3.1 (*7*–*9*) followed by primer trimming, adaptor trimming, and Phred quality score filtering (removing those with a score of <20) using Cutadapt v1.8.3 (*10*). Duplicate reads were removed using the Python script Dedup.py (*11*). The deduplicated FASTQ reads were assembled into contigs using the de novo assembler SPAdes v3.7.0 (*12*) using multiple k-mers. In addition to de novo assembly, read mapping was also performed with reference sequences using Geneious vR11.1.2.

References

1. Park GW, Chhabra P, Vinjé J. Swab sampling method for the detection of human norovirus on surfaces. J Vis Exp. 2017;120. PubMed https://doi.org/10.3791/55205

2. Liu P, Escudero B, Jaykus LA, Montes J, Goulter RM, Lichtenstein M, et al. Laboratory evidence of Norwalk virus contamination on the hands of infected individuals. Appl Environ Microbiol. 2013;79:7875–81. PubMed https://doi.org/10.1128/AEM.02576-13

3. Park GW, Williamson KJ, DeBess E, Cieslak PR, Gregoricus N, De Nardo E, et al. High hand contamination rates during norovirus outbreaks in long-term care facilities. Infect Control Hosp Epidemiol. 2018;39:219–21. PubMed https://doi.org/10.1017/ice.2017.274

4. Liang Y, Jin X, Huang Y, Chen S. Development and application of a real-time polymerase chain reaction assay for detection of a novel gut bacteriophage (crAssphage). J Med Virol. 2018;90:464–8. PubMed https://doi.org/10.1002/jmv.24974

5. Vega E, Barclay L, Gregoricus N, Williams K, Lee D, Vinjé J. Novel surveillance network for norovirus gastroenteritis outbreaks, United States. Emerg Infect Dis. 2011;17:1389–95. PubMed https://doi.org/10.3201/eid1708.101837

6. Montmayeur AM, Ng TF, Schmidt A, Zhao K, Magaña L, Iber J, et al. High-throughput next-generation sequencing of polioviruses. J Clin Microbiol. 2017;55:606–15. PubMed https://doi.org/10.1128/JCM.02121-16

7. Langmead B, Trapnell C, Pop M, Salzberg SL. Ultrafast and memory-efficient alignment of short DNA sequences to the human genome. Genome Biol. 2009;10:R25. PubMed https://doi.org/10.1186/gb-2009-10-3-r25

8. Langmead B, Wilks C, Antonescu V, Charles R. Scaling read aligners to hundreds of threads on general-purPositivee processors. Bioinformatics. 2019;35:421–32. PubMed https://doi.org/10.1093/bioinformatics/bty648

9. Langmead B, Salzberg SL. Fast gapped-read alignment with Bowtie 2. Nat Methods. 2012;9:357–9. PubMed https://doi.org/10.1038/nmeth.1923

10. Martin M. Cutadapt removes adapter sequences from high-throughput sequencing reads. EMBnet J. 2011;17:10–12.

11. Deng X, Naccache SN, Ng T, Federman S, Li L, Chiu CY, et al. An ensemble strategy that significantly improves de novo assembly of microbial genomes from metagenomic next-generation sequencing data. Nucleic Acids Res. 2015;43:e46. PubMed https://doi.org/10.1093/nar/gkv002

12. Bankevich A, Nurk S, Antipov D, Gurevich AA, Dvorkin M, Kulikov AS, et al. SPAdes: a new genome assembly algorithm and its applications to single-cell sequencing. J Comput Biol. 2012;19:455–77. PubMed https://doi.org/10.1089/cmb.2012.0021

Publisher: CDC; Journal: Emerging Infectious Diseases
Article Type: Research; Volume: 26; Issue: 8; Year: 2020; Article ID: 20-0346
DOI: 10.3201/eid2608.200346; TOC Head: Research

Appendix Table. Detection of CrAssphage and norovirus in stool and hand-rinse samples from norovirus outbreaks in long-term care facilities

				Stool specimen		Hand rinse sample	
Sample	Outbreak ID	Patient ID	Role	CrAssphage	Norovirus*	CrAssphage	Norovirus*
1	A	0587	Healthcare worker	Negative	Negative	Negative	Negative
2	E	0639	Healthcare worker	Negative	GI.4	Confirmed†	Negative
3	F	0629	Resident	Negative	GII.1†	Negative	GII.1†
4	F	0637	Resident	Confirmed†	GII.1†	Positive‡	GII.1†
5	E	0626	Healthcare worker	Confirmed†	GI.4	Confirmed†	Negative
6	G	0636	Resident	Confirmed†	GII.4 Sydney	Confirmed†	GII.4 Sydney
7	G	0635	Resident	Confirmed†	GII.4 Sydney	Confirmed†	Negative
8	D	0618	Healthcare worker	Negative	GII.4 Sydney	Confirmed†	Negative
9	D	0633	Resident	Confirmed†	GII.4 Sydney	Positive‡	GII‡
10	B	0600	Healthcare worker	Confirmed†	GII.4 Sydney	Confirmed†	Negative
11	B	0601	Healthcare worker	Confirmed†	Negative	Confirmed†	GII‡
12	B	0608	Resident	Confirmed†	Negative	Confirmed†	Negative
13	B	0605	Resident	Confirmed†	GII.4 Sydney	Positive‡	Negative
14	B	0609	Resident	Confirmed†	GII.4 Sydney	Positive‡	GII.4 Sydney
15	B	0611	Resident	Confirmed†	GII.4 Sydney	Confirmed†	GII‡
16	C	0644	Healthcare worker	Positive‡	GII.4 Sydney	Positive‡	Negative
17	C	0645	Healthcare worker	Negative	GII.4 Sydney	Confirmed†	GII‡
18	C	0630	Healthcare worker	Confirmed†	GII.4 Sydney	Negative	Negative
19	C	0616	Resident	Confirmed†	GII.4 Sydney	Negative	GII.4 Sydney
20	C	0634	Healthcare worker	Negative	Negative	Negative	Negative
21	H	0593	Resident	Confirmed†	GII.4 Sydney	Confirmed†	GII.4 Sydney
22	I	0596	Resident	Confirmed†	GII.4 Sydney	Confirmed†	GII.4 Sydney
23	J	0622	Healthcare worker	Negative	GII.6	Negative	Negative
24	J	0620	Healthcare worker	Negative	Negative	Negative	Negative
25	J	0621	Resident	Confirmed†	GII.6	Confirmed†	GII.6
26	K	0659	Resident	Positive‡	GII.6	Negative	Negative
27	K	0670	Healthcare worker	Negative	Negative	Negative	Negative
28	K	0658	Healthcare worker	Confirmed†	GII.6	Negative	GII.6
29	L	0660	Resident	Negative	GII.†	Negative	GII.†
30	L	0663	Healthcare worker	Negative	Negative	Negative	Negative

*Data published in Park et al (*3*).
†Sequence confirmed. Additional information in Figure 2 (https://wwwnc.cdc.gov/EID/article/26/8/20-0346-F2.htm).
‡Positive by real-time (reverse transcription) PCR assay.
